# Efficiency of Carbon Dioxide Fractional Laser in Skin Resurfacing 

**DOI:** 10.3889/oamjms.2016.062

**Published:** 2016-05-24

**Authors:** Andrej Petrov

**Affiliations:** Acibadem Sistina Clinical Hospital, Skopje, Republic of Macedonia

**Keywords:** CO_2_, laser, resurfacing, skin, synergistic effect

## Abstract

**AIM:**

The aim of the study was to confirm the efficiency and safety of the fractional CO_2_ laser in skin renewal and to check the possibility of having a synergistic effect in patients who besides carbon dioxide laser are treated with PRP (platelet-rich plasma) too.

**MATERIAL AND METHODS:**

The first group (Examined Group 1 or EG1) included 107 patients treated with fractional CO_2_ laser (Lutronic eCO_2_) as mono-therapy. The second group (Control Group or CG) covered 100 patients treated with neither laser nor plasma in the same period but subjected to local therapy with drugs or other physio-procedures under the existing protocols for treatment of certain diseases. The third group (Examined Group 2 or EG2) treated 25 patients with combined therapy of CO_2_ laser and PRP in the treatment of facial rejuvenation or treatment of acne scars.

**RESULTS:**

Patient’s satisfaction, in general, is significantly greater in both examined groups (EG1 and EG2) (p < 0.001). It was found the significant difference between control and examined group from the treatment in acne scar (Fisher exact two tailed p < 0.001). Patients satisfaction with the treatment effect in rejuvenation of the skin is significant (χ2 = 39.41; df = 4; p < 0.001). But, patients satisfaction from the treatment with HPV on the skin was significantly lower in examined group (treated with laser), p = 0.0002.

**CONCLUSION:**

Multifunctional fractional carbon dioxide laser used in treatment of patients with acne and pigmentation from acne, as well as in the treatment of scars from different backgrounds, is an effective and safe method that causes statistically significant better effect of the treatment, greater patients’ satisfaction, minimal side effects and statistically better response to the therapy, according to assessments by the patient and the therapist.

## Introduction

The laser is the acronym from Light Amplification by Stimulated Emission of Radiation. Main characteristics of laser light are the monochromatic character, collimation and coherent beam. The fractional carbon dioxide laser is used in the treatment of many dermatological diseases, as well as in several aesthetic procedures. CO_2_ laser has wave length 10600 nm. The advantage of CO_2_ laser is the non-selective target for the laser beam in the body, because main medium of absorption is water [1].

The role of minimally invasive treatments in dermatology is to achieve maximum result with the minimal thermal injury to the skin. Anderson and Parish published original theory for selective photo thermolysis [2].

Maintain developed selective laser thermal injury for skin remodelling resulting in superficial ablation of the skin, characterised by islands of speared skin, from which recovery after laser treatment is starting [3].

eCO_2_ laser is the powerful weapon in the treatment of many dermatological diseases- wrinkles, scars, dilated pores, stretch-marks, benign growths on the skin. Some of laser side effects are progressively decreased, like thermal injury and recovery time, also need for effective anaesthesia, risk of developing depigmentation and scars, persistent erythema and long-term avoiding of sun exposure. CO_2_ laser, therefore, is good opportunity for the rejuvenation of the skin. Even ablative laser resurfacing of the skin is safe and semi-invasive, however careful approach is necessary for adjusting treatment parameters for the minimalisation of the complication and optimisation of the results [4].

Fractional foto thermolysis is the method of laser skin resurfacing by producing microscopic thermal wounds on the surface of the skin, divided by the islands of speared, non-affected tissue from which tissue recovery is starting, avoiding side effects and prolonged recovery. In 2014 Kauvar showed that fractional laser procedure is giving excellent results in various clinical conditions, including photo-damaged skin, atrophic scars, surgical wounds and burn scars. However, efforts to produce massive fibroplasia in histological simples were non-consistent. A better understanding of histological changes during fractional resurfacing can help in better understanding of the process and optimisation of the results [5].

One of the biggest study in this field with 43000 patients showed that CO_2_ laser can be the adequate alternative to conventional surgery in rejuvenation procedures and acne scars treatment. Authors emphasise precise vapourisation of the tissue and minimal side effects as the main benefit [6].

Knowledge about skin ageing is substantially increased in past decade. Skin is losing elasticity during the time. Rejuvenation is treatment and art in same time. We are not treating only wrinkles; instead, all cosmetic concepts of the patients are needed [7].

The aim of the study was to confirm the efficiency and safety of the fractional CO_2_ laser in skin renewal and to check the possibility of having a synergistic effect in patients who besides carbon dioxide laser are treated with PRP (platelet-rich plasma) too.

## Material and Methods

The study was prospective-retrospective, randomised and was carried out at Acibadem Sistina Clinical Hospital, at the Department of Dermatovenerology, in the period from 2013 - 2015, with a total number of 232 patients included in the study. The patients were randomised into three groups.

The first group (Examined Group 1 or EG1) included 107 patients treated with fractional CO_2_ laser (Lutronic eCO_2_) as mono-therapy. The second group (Control Group or CG) covered 100 patients treated with neither laser nor plasma in the same period but subjected to local therapy with drugs or other physio-procedures under the existing protocols for treatment of certain diseases. The third group (Examined Group 2 or EG2) treated 25 patients with combined therapy of CO_2_ laser and PRP in the treatment of facial rejuvenation or treatment of acne scars. The mechanism is thrown the stimulation of the fibroblasts and induction of new collagen. Alfa granules of the fibroblasts are secreting various growth factors and activated aggregation of thrombocytes. These factors include VEGF, PDGF, IGF, and TGF which are controlling migration and proliferation and differentiation. They increase mitosis [8].

Before every treatment the patients filled out a written consent to the treatment and entry into the study, as well as an informative consent which informed them about all the effects of the treatment and they were introduced to the necessary information.

Inclusive criteria for entering the study are patients who have one of the following clinical conditions: acne and residual efflorescence of acnes, scars from different backgrounds, stretches, photo damaged skin, hyperpigmentation, xanthelasma, syringoma, viral warts.

The exclusionary criterion in the study is the use of oral retinoids in the period of 6 months prior to the laser treatment. Oral retinoids are powerful photosensitisation agents and in mutual use with laser therapy, they increase the possibility of adverse effects in the form of solar erythema and burns. There are also studies that analyse the use of retinoids as a factor that slows down the wound healing after the applied laser treatment. Furthermore, use of anticoagulation therapy, age under 18, presence of systemic diseases in the patient, using artificial light sources -solarium and using sauna during the treatment, and use of fillers – hyaluronan loaders 6 months before the treatment, or use of any tissue fillers regardless the time limit of application, pregnancy in female patients undergoing treatment of photo-exposed parts, herpes viral skin infections.

The treatment in the examined group of patients was carried out with laser- eCO_2_/Lutronic, in accordance with the existing protocols for laser treatment, depending on the type of lesion and its depth. During the laser treatment, protective eyeglasses should use both the patient and the doctor, due to protection from the laser beam’s effects on the eye (the most serious side effect). Before treatment with the CO_2_ laser, a local anaesthetic /Lidocaine/ is applied - in the form of a cream, to avoid the feeling of pain from the laser beam. After the end of treatment sessions, the patients underwent mechanical cooling of the treated area with ice. In the treatment of lesions, the combination of a static and dynamic module or a combination of the two modules is used.

The interventions are performed with different pulse energy, strength, density and number of passes in a single session, and also with a different number of sessions, depending on the protocols of a particular disease (acne scars require a minimum of three sessions; skin rejuvenation 3-4 sessions, viral warts 1-2 sessions of laser treatment). Patients were treated with different intensities of the laser beam. Three types of eCO_2_ radiation beams – of 120 nm, 300 nm, and/or 500 nm – were used. Various modules penetrate at the different depth into the skin. The modules 500 and 300 have an effect on the surface while the beam of 120 nm penetrates deeper into the skin, but the radial width (side penetration) of the laser beam, in this case, is lower.

Before and after the treatment the patients were photographed and digital photographs were a basis for assessing the effect of the treatment. Each patient individually filled out a questionnaire to assess the effects of the treatment, as well as a separate questionnaire for subjective evaluation of the adverse effects of the therapy (pain, redness, and pigmentations).

In the evaluation of digital photography, an improvement was independently assessed by the doctor and another dermatologist and expressed as a cumulative summary assessment. A questionnaire filled out by the patients and a digital photography with Molemax HD/Cosmetic module was used for the assessment of the results. All the patients in the study were digitally photographed with the Molemax system for the objectivity of the results. Patients were followed up for three and six months after the treatment and evaluated independently.

Patients in the second group (Control Group or CG) undergone therapeutic modalities depending on the diagnosis of the disease, and according to the existing protocols for certain diseases. There was one of the three elective standard therapeutic non-laser treatments to compare, under the protocols for a treatment of certain diseases, widely used in everyday ambulatory practice. For patients with acne scars and patients who undergone rejuvenation, the comparison was made with retinoids, topically applied to the skin. For patients with viral diseases of the skin, the comparison was made with standard, the most widely used method for treatment of these diseases - cryotherapy (freezing therapy, cryosurgery).

In the treatment of the third group of patients (Examined Group 2) two methods were used: treatment with eCO_2_ laser and PRP. Two weeks after the last laser treatment 25 patients took two sessions of PRP injections with a derma roller type 0.5 and 1 mm, and 30G needles. The distance between two treatments with platelet-rich plasma (pure plasma) was two weeks. Within the PRP method owns platelet-rich plasma with a high growth factor is injected. It is actually a stimulation of fibroblasts and induction of a new collagen. It means an injection of a kind of endogenous filler which is a natural part of the body. Since it comes from the patient, it does not cause a risk of allergic reactions or infection. Before starting the treatment the patient blood was drawn, processed and centrifuged in our haematological laboratory at the Acibadem Sistina Clinical Hospital, resulting in purified platelet-rich plasma, about 6 ccm. Before applying platelet-rich plasma to the skin or the capillitium, a local anaesthetic in the form of cream - Emla (Lidocaine), which needs to remain about 40 min to 1 hour, was applied to the skin and then removed to allow painless treatment. Injection of the plasma into the skin or capillitium was performed with a thin insulin needle or derma roller 0.5 to 1mm size or a special applicator - Dermapen with different sizes of needles (penetration depth) of 0.5, 0.75 or 1mm.

The intervention is after two weeks. Full effects come after 1.5-2 months, and they usually persist 18 months after the treatment. We used purified plasma in the study that leaves no colouring on the skin after the application. Patients endure the intervention without much discomfort.

1. The study differentiated three major subgroups of patients with statistical significance in the examined group treated with the laser as mono-therapy. The first subgroup is patients with acne scars, the second large subgroup are patients treated for rejuvenation of the skin. They were compared with the patients who used a topical retinoid - Adapalene in the therapy (control group of 66 patients) and with patients undergoing laser treatment combined with PRP method - Examined Group 2 (25 patients). Within the group of patients with acne scars effects in different subgroups too were evaluated, depending on the type of acnes.

In patients with viral skin growths (the third subgroup of 54 patients), there was the comparison between laser therapy and other therapeutic modalities under the protocols, primarily with widely used method - cryotherapy.

2. Some of the patients were treated for other indications according to the inclusive criteria which were compared respectively and given as a review.

3. All treated patients were monitored for the effectiveness of laser therapy and the side effects of it.

### Statistical analysis

For a statistical analysis of data obtained during the survey a base in the statistical program SPSS for Windows 13.0 was created. During the computer analysis, statistical methodologies were used as follows:

- Distribution of frequencies (absolute and relative representation) to present the categorical variables;
- Descriptive methods (measures of central tendency - average, median, minimum, maximum values to present the quantitative variables;
- To test the significance of differences between the analysed groups non-parametric (Chi-square test, Fisher exact test, Mann-Whitney U test), and parametric tests (t-test for independent samples, Analysis of Variance) were used depending on the nature of the data i.e. their distribution;
- As a level of significance or importance was taken a value of p <0.05, and as a highly significant value of p <0.01.

### Results

Distribution of the investigated patients between all three groups is shown in Table 1.

**Table 1:** Age related distribution of the patients between all 3 groups

Age groups	CG N = 100	EG1 N = 107	EG2 N = 25
**> = 19**	28 (28%)	12 (11.21%)	3 (12%)
**20 – 29**	22 (22%)	31 (28.97%)	8 (32%)
**30 – 39**	25 (25%)	38 (35.51%)	6 (24%)
**40 – 49**	13 (13%)	20 (18.69%)	6 (24%)
**50 >**	12 (12%)	6 (5.61%)	2 (8%)

Tested differences	CG vs. EG1 χ^2^=13.9, df = 4, p = 0.008** CG vs. EG1PRP Fisher exact two tailed p = 0.3 EGL vs. EG1PRP Fisher exact two tailed p = 0.3		

**Figure 1:**
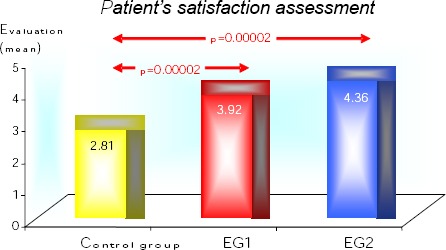
*Effect of the treatment between 3 groups- patients satisfaction assessment*.

Patient’s satisfaction assessment in a control group, EG1, and EG2 is shown in Fig. 1. We can see that patient’s satisfaction is significantly greater in both experimental groups (p < 0.001).

Distribution of the answers assessment of the subjective satisfaction from the treatment in acne scar patients is shown in Table 2. It was found the significant difference between control and experimental group (Fisher exact two tailed p < 0.001).

**Table 2:** Distribution of the answers „assessment of the subjective satisfaction from the treatment in acne scar patients”

Acne scars patient assessment of the satisfaction from the treatment	Control group (CG) N = 46	Examined group (laser) (EG) N = 28
1 - Not satisfied	6 (13.04%)	0
2 - Partially not satisfied	20 (43.48%)	0
3 – Neutral	8 (17.39%)	3 (10.71%)
4 - Partially satisfied	8 (17.39%)	9 (32.14%)
5 - Totally satisfied	4 (8.7%)	16 (57.14%)

Tested differences	CG vs. EG Fisher exacts two tailed p < 0.001	

Patients satisfaction with the treatment effect in the rejuvenation of the skin is shown in Table 3. Differences between control group (CG) and experimental group (EG) are significant (χ2 = 39.41; df = 4; p < 0.001).

**Table 3:** Patients satisfaction with the treatment effect in rejuvenation of the skin

Assessment of the effect of the treatment in patients for rejuvenation?	CG N = 36	EG (laser) N = 21
1- Not satisfied	7 (19.44%)	0
2- Partially not satisfied	15 (41.67%)	0
3- Neutral	12 (33.33%)	3 (14.29%)
4- Partially satisfied	2 (5.56%)	11 (52.38%)
5- Totally satisfied	0	7 (33.33%)
Tested differences	CG vs. EG (laser) χ^2^= 39.41; df = 4; p < 0.001	

**Figure 2:**
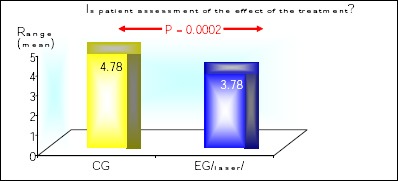
*Patients satisfaction from the treatment in patients with HPV on the skin*.

Patients satisfaction from the treatment with HPV on the skin was significantly lower in the experimental group (treated with laser), p = 0.0002 (Fig. 2).

**Figure 3:**
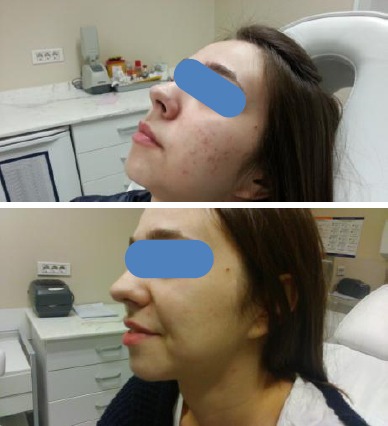
*a) Patient with acne scars before treatment with CO_2_ laser; b) The same patient from the previous picture after 4 treatments with CO_2_ laser*.

Patient with acne scars before and after treatment with CO_2_ laser is shown in Fig. 3.

**Figure 4:**
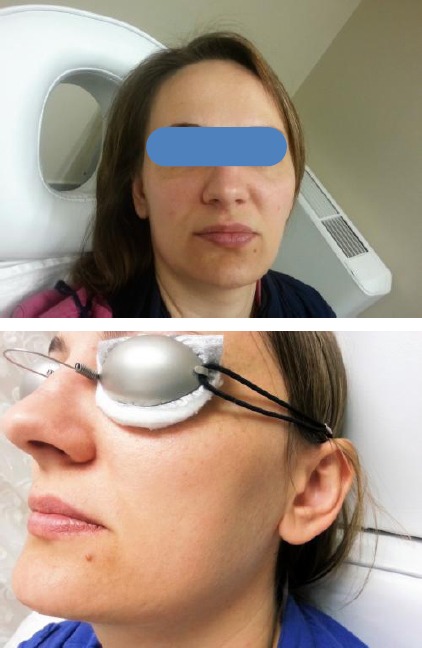
*a) Patient for rejuvenation before treatment; b) Same patient from the previous picture after 3 sessions of CO_2_ laser*.

One patient for rejuvenation before and after 3 sessions of CO_2_ laser treatment is shown in Fig. 4.

**Figure 5:**
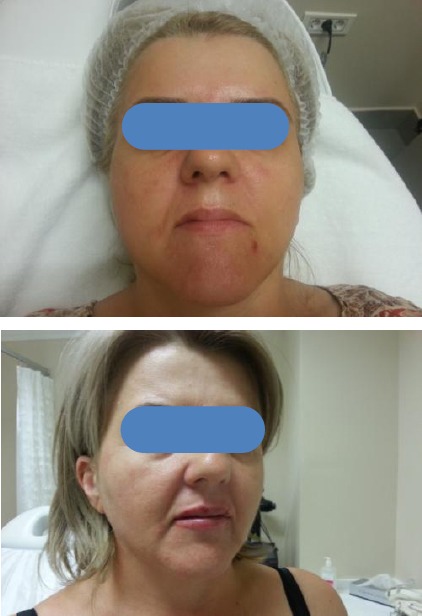
*a) Patient treated with plasma and CO_2_ laser before treatment; b) the Same patient from picture 7, after 3 sessions of CO_2_ laser and 2 sessions of PRP, during lip augmentation*.

Patient before treatment and after 3 session’s treatment with CO_2_ laser and 2 session’s treatment with PRP, during lip augmentation, is shown in Fig. 5.

In all three subgroups of the laser examined group (acnes, rejuvenation, viral warts) there are no statistically significant mutual differences in relation to the laser treatment, i.e. it is equally efficient. The laser treatment with carbon dioxide laser can be safely used in combination with other aesthetic treatments such as platelet-rich plasma, botox, fillers and other aesthetic procedures. The combined effect of the laser treatment and platelet-rich plasma is rated by the patients with statistically significant greater subjective satisfaction and effects in terms of post-treatment pigmentations, compared to the control group.

The combined laser treatment and platelet-rich plasma, versus laser monotherapy, shows greater cumulative assessment of the effect of applied treatment in our study, but without statistical significance, i.e. it is not significant.

### Discussion

A group of authors made revue on 20 papers published between 2008 and 2013 for acne scars treatment. Histology findings confirmed the same conclusion as in our study about the effect of the fractional laser [9].

Clinical and histological studies are illustrating 50-80% efficacy of CO_2_ laser treatment in atrophic acne scars of the face. Variation of the effects is probably due to differences in the type and extensity of the scars [10].

In the treatment of skin rejuvenation, the laser treatment is effective and safe, causing statistically significant higher satisfaction in relation to the control group of patients. Satisfaction with the treatment in patients undergoing rejuvenation is greater than in any of the examined groups.

According to the group of American authors laser resurfacing of the skin is powerful weapon in the treatment of many conditions as various wrinkles, dilated pores, benign skin growths and improving the elasticity of the skin or texture [11].

The fractional carbon dioxide laser is safe to use in a treatment of viral diseases of the skin and mucous membranes, condylomas and warts, without distinct side effects and discomfort in the treated patients. However, the effect of the therapy and patients’ satisfaction, and also evaluation by the doctors, in our study is statistically lower in relation to the control group of patients.

Groups of an author are referring opposite conclusions in the randomized study on the larger group of patients. They are showing two times better effect after laser treatment comparing with freezing therapy and the statistically lower number of recidivism of condylomas [12].

However, CO_2_ laser for condyloma can be better tolerate regarding burning sensation and after treatment pigmentation because precise laser ablation of the tissue. The results are compatible with a data from literature [13, 14].

The combination of different techniques FACELIFT which is the acronym from Augmentation of Collagen and Elastin using Lasers, Fillers and Topicals, is the effective alternative of face-lift [15].

At the same time, the combined laser therapy with PRP is associated with greater patients’ satisfaction with the treatment and with significantly greater satisfaction with the appearance of pigmentation after the treatment versus laser mono-therapy.

The conclusion of the study, based on the results, is that multifunctional fractional carbon dioxide laser used in treatment of patients with acne and pigmentation from acne, as well as in the treatment of scars from different backgrounds, is an effective and safe method that causes statistically significant better effect of the treatment, greater patients’ satisfaction, minimal side effects and statistically better response to the therapy, according to assessments by the patient and the therapist.
